# Naturally Occurring Feline Cancers in Comparative Oncology: Translational Insights from Oral Squamous Cell Carcinoma and Mammary Carcinoma

**DOI:** 10.3390/cancers18132136

**Published:** 2026-07-01

**Authors:** Yinghua Wang, Jillian Elizabeth Yant, Xuan Pan

**Affiliations:** 1 Department of Medical Sciences, School of Veterinary Medicine, University of Wisconsin, Madison, WI 53706, USA; 2 Carbone Cancer Center, University of Wisconsin, Madison, WI 53705, USA; 3 Wisconsin Blood Cancer Research Institute, University of Wisconsin, Madison, WI 53705, USA

**Keywords:** comparative oncology, spontaneous feline tumors, feline oral squamous cell carcinoma, feline mammary carcinoma, head and neck squamous cell carcinoma, breast cancer

## Abstract

**Background:** Comparative oncology uses naturally occurring cancers in companion animals to study tumor biology and therapeutic responses relevant to human cancer. Spontaneous feline tumors are increasingly recognized as useful comparative models because they arise in immunocompetent hosts, develop under shared environmental exposures, and can reproduce selected clinical, histopathologic, molecular, and therapeutic features of human malignancies. **Methods:** This review compares feline oral squamous cell carcinoma (FOSCC) with human head and neck squamous cell carcinoma (HNSCC), and feline mammary carcinoma (FMC) with human breast cancer, emphasizing shared pathologic, molecular, tumor microenvironment, and therapeutic features. **Results:** Recent immunohistochemical, genomic, transcriptomic, and biomarker studies have identified shared features between feline and human cancers. FOSCC resembles human HNSCC through aggressive local invasion, histologic features, therapeutic resistance, and recurrent alterations of *TP53*, *MYC*, and *PTEN*. FMC shows strong overlap with aggressive human triple-negative breast cancer, including reduced hormone receptor expression, recurrent *TP53*, *PIK3CA*, and CXCL12/CXCR4 signaling alterations, and tumor microenvironment features involving immune-checkpoint, inflammatory, and angiogenic pathways. FOSCC clinical trials and emerging clinical investigations into FMC treatments further support the use of cats for translational therapy evaluation. **Conclusions:** FOSCC and FMC are promising comparative oncology models for human HNSCC and aggressive breast cancer, respectively. Future multicenter studies incorporating standardized tumor classification and grading, predefined stratification criteria, and clinically meaningful endpoints will be essential to strengthen their translational value.

## Simple Summary

Comparative oncology studies naturally occurring cancers in pet animals to better understand similar cancers in people and improve treatment for both. Cats are becoming uniquely helpful because they develop cancers naturally, have intact immune systems, share many environmental exposures with humans, and often show aggressive disease patterns that resemble human cancers. In this review, we focus on two common feline cancers, oral squamous cell carcinoma and mammary (breast) carcinoma, which share important clinical and biological features with human head and neck cancer and an aggressive form of human breast cancer. These similarities suggest that studying cancer in cats can help researchers better understand how similar cancers develop in people and test new treatments in settings that more closely reflect real-world disease.

## 1. Introduction

In the United States, an estimated 89 million pet dogs and 62 million pet cats reside in households [1]. These companion animals share many environmental exposures with humans and often develop cancers that resemble human disease in genetic pathways, histology, clinical presentation, disease outcomes, and responses to treatment [2,3,4]. Comparative oncology uses biologic and clinical insights from naturally occurring cancers in companion animals to improve understanding of cancer biology, support biomarker discovery, and advance therapeutic development in humans [2,3]. This approach is closely aligned with the concept of “One Health” [5,6,7], which emphasizes the bidirectional exchange of knowledge between veterinary and human medicine to improve health outcomes across species [8].

**Figure 1:**
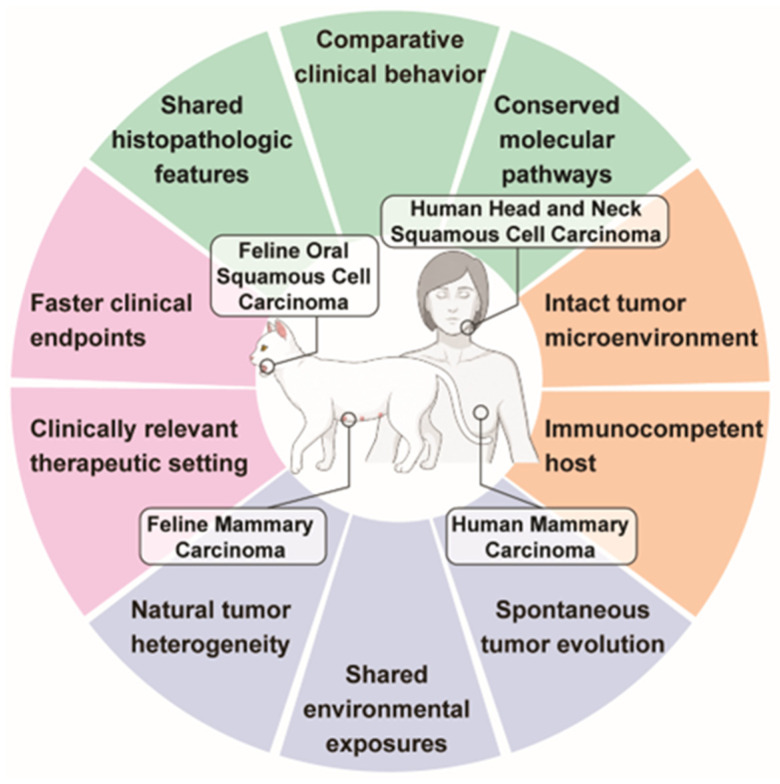
Key advantages of spontaneous feline cancers in comparative oncology. Spontaneous feline cancers develop in a real-world disease context, preserve host–tumor interactions, and often reach clinical endpoints more rapidly than comparable human cancers. FOSCC and FMC are highlighted as representative examples that recapitulate important aspects of human head and neck squamous cell carcinoma and mammary carcinoma, respectively, in their clinical behavior, histopathology, genomic alterations, and dysregulated signaling pathways. These features support the value of naturally occurring feline cancers as comparative oncology models for biomarker discovery, translational therapeutic studies, and evaluation of treatment response in spontaneous disease.

Spontaneously arising feline tumors offer several advantages over in vitro systems and conventional rodent models (Figure 1). Unlike experimentally induced or implanted tumors, cancers in cats develop in an immunocompetent host and can better reflect the natural history and biologic heterogeneity of human cancers [9,10,11,12,13,14]. Moreover, because cats have shorter life spans than humans and many feline cancers progress rapidly, longitudinal clinical and biologic data can often be collected over a shorter time frame [4,15]. Complementing these features, advances in molecular characterization have enabled the evaluation of feline tumors with greater resolution, further strengthening their translational relevance [4,16]. Recent studies have shown that feline cancers share many genetic and molecular similarities with human cancers, including frequent alterations in cancer-associated genes and pathways [12,16,17,18]. For example, targeted sequencing of 493 feline tumor samples and matched normal tissues across 13 tumor types revealed strong similarities between the feline and human oncogenomes [16]. *TP53* was the most frequently mutated gene in feline tumors, with a prevalence similar to that reported in human cancer datasets [16,19,20,21,22]. Feline tumors also showed recurrent copy-number gains in *MYC* and losses in *PTEN* and *FAS*, alterations that are broadly consistent with patterns observed across human cancers [16,23,24]. Histologic evidence further supports the comparative value of feline cancers. Across several tumor types, including feline oral squamous cell carcinoma (FOSCC), feline mammary carcinoma (FMC), pulmonary carcinoma, and intestinal carcinoma, feline tumors show features similar to those observed in humans, including comparable tissue architecture, differentiation patterns, cytologic atypia, stromal responses, and invasive growth behavior [25,26,27,28]. Together, these genetic, molecular, and histologic parallels support the use of naturally occurring feline cancers as comparative oncology models.

Among feline cancers, FOSCC and FMC are particularly promising comparative models for HNSCC and human mammary carcinoma, respectively [4,13,26,29,30,31]. This review examines FOSCC and FMC within the context of comparative oncology, emphasizing their histopathologic and molecular parallels with human cancers and their potential value for biomarker discovery, therapeutic evaluation, and translational research. The goal is not to suggest a simple one-to-one correspondence between feline and human malignancies but to critically evaluate where the comparative evidence is strongest, where important biologic differences remain, and what additional studies are needed to strengthen the contribution of feline oncology to human cancer research.

## 2. FOSCC and Human HNSCC

FOSCC is the most common oral malignancy in cats and the fourth most common feline cancer overall [30,32,33,34,35]. It arises from the squamous epithelium of the oral cavity and most commonly affects the gingiva, tongue, sublingual region, and tonsils [32,33]. Clinically, FOSCC is highly locally invasive and can cause extensive destruction of surrounding soft tissues and bones, leading to pain, difficulty swallowing, excessive salivation, weight loss, and reduced quality of life [33,36,37]. Although metastasis is generally uncommon at initial diagnosis, regional lymph node and pulmonary metastases can occur in advanced disease. This is particularly true for tonsillar FOSCC, which often shows more aggressive biologic behavior with a high metastatic rate [38,39]. Despite multimodal treatment approaches, including surgery, radiation therapy, chemotherapy, and immunotherapeutic strategies [40,41,42], the prognosis remains poor, with reported 1-year survival rates of less than 10% [32,37]. In humans, HNSCC is among the most common cancers worldwide and remains a major cause of cancer-related mortality [43,44]. Many patients present with locally advanced disease, and the overall 5-year survival rate is approximately 50% [44,45]. FOSCC shares several important clinical features with human HNSCC, including aggressive local invasion with destruction of surrounding tissues, relatively late metastatic progression, and limited response to conventional therapies [43]. In both species, tumors are often diagnosed at advanced stages, contributing to poor outcomes and highlighting the need for improved early detection and more effective therapeutic strategies [33,43].

### 2.1. Viral Etiology

Human HNSCC is commonly divided into two major etiologic subtypes: HPV-positive and HPV-negative disease. HPV-positive HNSCC is driven by infection with high-risk HPV strains, whereas HPV-negative HNSCC is more often associated with carcinogen exposure, particularly tobacco and alcohol usage [46,47]. These subtypes differ substantially in biology and clinical behavior. Compared with HPV-positive disease, HPV-negative HNSCC is generally associated with shorter survival, higher rates of local recurrence, and greater risk of distant metastasis [48,49]. Current evidence suggests that most FOSCC cases more closely resemble HPV-negative HNSCC than HPV-positive HNSCC [16,30,31,50,51]. Although *Felis catus* papillomavirus (FcaPV) DNA has been detected in some FOSCC cases, the reported detection rate varies widely across studies, from rare or absent detection to approximately 47% in selected cohorts [16,30,31,50,51,52,53,54,55]. This variation is difficult to interpret because studies differ in cohort size, geographic origin, tumor site, tissue preservation, and detection method. Earlier PCR-based studies, often using broad or non-type-specific primers on formalin-fixed paraffin-embedded tissue-derived DNA, reported rare or absent viral detection, and virome sequencing detected FcaPV in only a small minority of FOSCCs [31,52]. In contrast, studies using FcaPV type-specific PCR assays have reported higher detection rates [53,54,55]. One study detected FcaPV2 DNA in 46.9% of feline SCC cases from Taiwan but in only 8.6% of cases from Japan, suggesting possible geographic variation [54]. Overall, the wide range of reported FcaPV detection rates likely reflects biologic and geographic variation, as well as differences in assay sensitivity. However, detection of viral DNA alone does not prove that FcaPV drives tumor development. Stronger evidence of active viral involvement would require viral oncogene expression, a high viral load in tumor tissue, and viral localization within tumor cells [51,53,54,55]. Current evidence therefore supports FcaPV infection as a plausible contributor in a subset of FOSCCs, while most FOSCC cohorts not selected for viral positivity remain more consistent with HPV-negative human HNSCC biology [31,51]. At the molecular level, FOSCC also shares several alterations with HPV-negative HNSCC, including copy-number gains in *MYC* and copy-number losses in *PTEN* and *FAS* [12,16,20,21,56,57]. Together, virologic and molecular evidence support FOSCC as a closer comparative model for HPV-negative HNSCC than for HPV-positive disease [30,31,51,58,59].

### 2.2. Risk Factors

Risk factors for human HNSCC include chronic inflammation, HPV infection, periodontitis, and poor oral hygiene, with tobacco smoking and alcohol consumption representing the predominant drivers [43,60,61,62]. These factors create a sustained inflammatory and carcinogenic environment that promotes squamous epithelial transformation and tumor progression. In cats, exposure to environmental tobacco smoke has been associated with an increased risk of FOSCC, potentially because tobacco-derived carcinogens accumulate on the fur and are subsequently ingested during grooming [63,64]. This finding parallels the role of tobacco exposure in human HNSCC and supports the concept that shared carcinogens may contribute to oral squamous carcinogenesis across species. Additional environmental and lifestyle factors have also been associated with FOSCC, including ectoparasite-control products, flea-collar use, and dietary variables [35,63,64]. Flea-collar exposure has been associated with an approximately 5-fold increased risk of FOSCC, whereas high consumption of canned food and canned tuna has been associated with approximately 3.6-fold and 4.7-fold increased risks, respectively [63]. Collectively, these findings indicate that both human HNSCC and FOSCC arise in the context of chronic environmental exposures and sustained inflammatory stimuli.

### 2.3. Histopathologic Similarities Between Feline and Human Oral Squamous Cell Carcinoma (OSCC)

FOSCC closely resembles human OSCC histologically. In both species, the predominant histopathologic feature is invasive squamous differentiation, in which malignant epithelial cells form nests, islands, cords, or sheets that infiltrate the underlying stroma. FOSCC and human OSCC also show variable degrees of keratinization, cytologic atypia, nuclear pleomorphism, and mitotic activity. These epithelial changes are often accompanied by a stromal response characterized by desmoplasia, fibrosis, and mixed inflammatory cell infiltration [25,33,65,66]. In FOSCC, the invasive tumor component commonly consists of small clusters, cords, or trabeculae of neoplastic cells extending into the submucosa and deeper tissues, closely mirroring the infiltrative growth pattern described in human OSCC [25,33]. Both diseases are also characterized by marked local invasion, including extension into adjacent soft tissues and bone, consistent with their clinically aggressive behavior [33,37]. In addition to conventional histologic patterns, FOSCC may display variants such as verrucous and papillary forms, which correspond to recognized morphologic subtypes within the human head and neck cancer spectrum [33,67].

Despite these strong histopathologic similarities, feline-specific grading and classification systems for FOSCC remain incompletely standardized. Several studies have applied human-derived grading schemes, including Anneroth’s and Bryne’s systems, and have described histologic subtypes such as conventional, verrucous, papillary, acantholytic, and adenosquamous variants [68,69]. However, the prognostic relevance of these grading approaches and subtypes has not been consistently validated in cats. As a result, FOSCC currently lacks a universally accepted feline-specific histopathologic grading framework. Establishing standardized criteria that integrate histology with clinical outcomes, molecular features, and therapeutic response would improve case stratification, enhance reproducibility across studies, and strengthen the translational value of FOSCC as a comparative model for human OSCC/HNSCC.

### 2.4. Shared Molecular Pathways and Oncogenic Drivers in FOSCC and Human HNSCC

Genomic and transcriptomic profiling has revealed substantial overlap between FOSCC and human HNSCC at both the mutational and gene-expression levels [12,34,70], as summarized in Table 1. These shared alterations span several biological processes, including tumor suppressor disruption, invasion-associated transcriptional programs, inflammatory and receptor-mediated signaling, angiogenesis, and developmental pathway dysregulation. In HNSCC, *TP53* is the most consistently altered gene, with mutations reported in approximately 70% of cases and recognized as key events in tumor initiation and progression [71,72,73]. *TP53* alterations are also prominent in FOSCC, although reported frequencies vary across cohorts and sequencing platforms. Whole-exome sequencing identified *TP53* mutations in 12 of 42 FOSCC cases (29%), while a smaller cohort detected *TP53* mutations in 4 of 6 cases [16,34]. In a recent targeted sequencing study, 24 of 34 tumors (71%) contained predicted consequential *TP53* variants [12]. These genetic alterations are further supported by transcriptomic evidence of TP53 pathway disruption in FOSCC, including reduced expression of the p53 target gene *CDKN1A* [34].

FOSCC and HNSCC share activation of epithelial–mesenchymal transition (EMT)-associated programs. EMT promotes tumor cell plasticity, local invasion, metastasis, and treatment resistance, making it highly relevant to the aggressive behavior of both diseases [74,75,76,77]. In human OSCC/HNSCC, EMT is associated with poor prognosis and treatment resistance and is marked by increased expression of transcriptional regulators such as *ZEB1*, *ZEB2*, and *TWIST1* [74,78]. Transcriptomic profiling of FOSCC has shown enrichment of a similar EMT-associated program, including increased expression of *ZEB1*, *ZEB2*, and *TWIST1* [34,79,80]. This shared EMT signature suggests that FOSCC and HNSCC may use similar mechanisms to promote invasion, metastasis, and therapy resistance, supporting the relevance of FOSCC for studying tumor plasticity in a comparative oncology context.

Inflammation-associated signaling represents another shared feature that may contribute to invasion and treatment resistance in FOSCC and human HNSCC. Transcriptomic profiling of FOSCC has shown enrichment of IL-6/JAK/STAT, TNF/NF-κB, hypoxia, and related inflammatory pathways [34]. Consistent with this pathway-level evidence, STAT3 activation has also been detected in FOSCC-derived cell lines [81]. Pharmacologic STAT3 inhibition in pet cats with FOSCC achieved a 35% disease control rate, further supporting the potential therapeutic relevance of this pathway [41]. In HNSCC, IL-6-mediated activation of JAK/STAT3 signaling promotes tumor growth, immune evasion, therapeutic resistance, and poor clinical outcomes [82,83,84,85,86]. EGFR signaling can activate the JAK/STAT3 signaling pathway [87], and EGFR overexpression has been reported in approximately 69–100% of FOSCC cases and 90% of HNSCC cases and is associated with increased proliferation, invasion, and treatment resistance [34,88,89,90].

FOSCC and HNSCC also share signaling pathways that regulate angiogenesis, tumor growth, and invasion. In HNSCC, COX-2 promotes tumor progression in part through prostaglandin-mediated signaling and regulation of angiogenic mediators such as *VEGF* [91,92,93,94,95,96]. A similar pattern has been reported in FOSCC, where COX-2 overexpression occurs in a subset of cases and is often associated with VEGF expression [97,98,99,100]. Aberrant WNT/β-catenin signaling has also been described in both HNSCC and FOSCC [25,101,102,103]. Because WNT/β-catenin regulates development, epithelial homeostasis, and cell fate, its dysregulation may contribute to tumor growth and invasion in both diseases [25,101,102,103,104].

Taken together, these findings indicate that FOSCC and HNSCC share alterations in TP53-mediated tumor suppression, EMT-associated invasion, STAT3-mediated inflammatory signaling, COX-2/VEGF-associated angiogenesis, and WNT/β-catenin developmental signaling. This molecular similarity provides a mechanistic rationale for using FOSCC as a spontaneous comparative model of selected pathway-driven mechanisms in HNSCC, particularly invasion, metastasis, and therapeutic resistance.

**Table 1:** Shared molecular features between FOSCC and human HNSCC.

Molecular Feature	Human HNSCC	FOSCC
*TP53*	Loss-of-function mutation of *TP53* is associated with loss of genome surveillance, impaired apoptosis and cell-cycle arrest, with mutations reported in approximately 70% of cases [71,72,73,105,106].	*TP53* mutations have been reported in up to 71% of FOSCC tumors [12,16,34].
*MYC*	MYC overexpression, with an estimated prevalence of 12% in HNSCC, is linked to aggressive behavior, poorer survival, and therapeutic resistance, particularly in HPV-negative tumors [107,108].	MYC expression is increased in FOSCC tissue, and recent whole-exome sequencing identified *MYC* copy-number gain in 31% of FOSCCs [16,25,34].
*PTEN*	In HNSCC, *PTEN* loss or inactivation promotes cell proliferation, migration, and survival, with mutations detected in ~10% of patients [109,110,111,112].	*PTEN* copy-number loss was identified in 24% of FOSCCs [16].
*FAS*	FAS pathway dysregulation contributes to apoptosis resistance and immune escape in HNSCC [113,114,115].	*FAS* copy-number loss was identified in 21% of FOSCCs [16].
*CDKN2A*	Loss-of-function mutation of *CDKN2A* has been identified as a prognostic biomarker in HNSCC, associated with poor overall survival and increased risk of metastasis [116,117,118].	*CDKN2A* inactivation has been reported in FOSCC [50,119].
EMT-related pathways	In HNSCC, upregulated EMT contributes to invasion, metastasis, poor prognosis, and treatment resistance [77,78].	FOSCC RNA-seq shows enrichment of EMT-related pathways [34,79,80].
IL-6/JAK/STAT3	In HNSCC, hyperactivated IL-6/JAK/STAT3 signaling promotes tumor growth, immune evasion, invasion, treatment resistance, and poor prognosis [82,83,84,85,86].	The IL-6/JAK/STAT pathway is enriched in FOSCC [41,81].
EGFR signaling	*EGFR* is up to 90% overexpressed in human HNSCC and is associated with proliferation, invasion, and treatment resistance [89,90,120].	*EGFR* is overexpressed in 69–100% of FOSCC cases and is associated with tumor aggressiveness [34,39,88].
*COX-2* and *VEGF*	In HNSCC, upregulation of *COX-2* and *VEGF* is linked to angiogenesis, lymph node metastasis, tumor progression, and poorer survival outcomes [93,94,95,96,121,122].	*COX-2* overexpression has been reported in approximately 33% of FOSCC cases, with VEGF colocalization [97,98,99,100,123].
WNT/β-catenin	WNT/β-catenin signaling is upregulated in HNSCC and contributes to tumor progression, invasion, and treatment resistance [101,102,103,104].	Upregulation of WNT pathway components has been reported in FOSCC [25].

FOSCC, feline oral squamous cell carcinoma; HNSCC, head and neck squamous cell carcinoma; OSCC, oral squamous cell carcinoma; HPV, human papillomavirus; EMT, epithelial–mesenchymal transition.

### 2.5. Translational and Clinical Trial Insights of FOSCC

FOSCC is highly aggressive and progresses rapidly after diagnosis. The median survival time after diagnosis of FOSCC is approximately 66 days, reflecting the highly aggressive nature of the disease [38,124,125]. This short clinical course allows treatment response and tumor-associated biologic changes to be evaluated relatively quickly, facilitating translational studies that are difficult to conduct over comparable time frames in human patients [30,42,59].

Targeted therapeutic clinical trials in FOSCC have demonstrated the feasibility of using this disease for translational treatment studies. In a phase I clinical trial of systemic cyclic STAT3 decoy oligonucleotide (CS3D) in 20 pet cats with FOSCC, treatment was well tolerated, with no dose-limiting toxicity observed at 10 mg/kg, and 7 of 20 cats showed either partial response or stable disease [41]. Notably, tumors from responding cats showed distinct immunologic features, including increased intratumoral PD-1 expression, suggesting that STAT3 inhibition may be associated with tumor immune environment responses [41]. Additional studies have evaluated FOSCC in the context of therapeutic approaches relevant to human HNSCC. In human HNSCC, cisplatin-based chemoradiotherapy is commonly used, although systemic toxicity can limit completion of planned treatment regimens, highlighting the need for better-tolerated therapeutic strategies [126,127]. In a retrospective study, cats with FOSCC treated with accelerated radiation and concurrent carboplatin, follow-up treatment with toceranib phosphate, a multi-target tyrosine kinase inhibitor, did not significantly improve overall survival or progression-free interval [40]. This finding suggests that empiric multi-kinase inhibition with toceranib may have limited benefit in biologically unselected cats with FOSCC and highlights the need for biomarker-guided patient selection. Another prospective feasibility study evaluated ultrasound- and microbubble-mediated bleomycin delivery in six cats with FOSCC as a site-specific drug-delivery strategy [128]. In this approach, bleomycin is given systemically, followed by intravenously administered microbubbles that circulate through the tumor vasculature and are activated locally by ultrasound [128]. This ultrasound–microbubble interaction can increase vascular permeability and temporarily permeabilize tumor-cell membranes, potentially improving bleomycin uptake within the tumor without increasing the systemic drug dose [129,130,131,132]. Although antitumor responses were limited, the study demonstrated technical feasibility and acceptable tolerability, supporting further optimization of this delivery strategy before broader translational application.

Metabolic targeting strategies have also been evaluated in FOSCC. In HNSCC, polyamine metabolism is an active therapeutic target because squamous carcinoma cells require polyamines for proliferation and tumor regrowth during radiotherapy [133,134]. Difluoromethylornithine (DFMO) inhibits polyamine synthesis and suppresses tumor growth in mouse models, but its efficacy can be limited when tumor cells compensate by increasing polyamine uptake from outside the cell [135,136,137]. To address this limitation, a phase I/II feline study combined DFMO with the polyamine transport inhibitor MQT 1426 in cats with FOSCC, thereby targeting both polyamine synthesis and uptake [138,139,140]. Serial tumor biopsies showed reduced intratumoral polyamine levels, confirming that the treatment affected its intended metabolic pathway [138]. Although clinical responses were limited, with partial responses in two cats and stable disease in six cats, the combination was feasible and provided important safety and proof-of-mechanism data [138]. These findings support further investigation of dual polyamine blockade as a translational strategy relevant to HNSCC.

Collectively, these studies support FOSCC as a clinically relevant comparative model for aggressive HNSCC. FOSCC shares important features with human HNSCC, including advanced-stage presentation, marked local invasion, limited response to standard therapies, p53 dysfunction, EMT activation, and inflammatory signaling [34,42,43,81,85,86,96,97,98,99,100,123]. Because FOSCC arises spontaneously, progresses rapidly, and has limited standard treatment options, it provides a practical setting for evaluating new therapeutic strategies relevant to both feline OSCC and human HNSCC. These features support FOSCC as a translational model for studying HNSCC biology and treatment response.

## 3. FMC and Human Mammary Carcinoma

FMC is the third most common neoplasm in cats, accounting for approximately 11–35.5% of all feline tumors [141,142]. The annual incidence has been estimated at approximately 230 cases per 100,000 cats in Sweden [143]. Unlike canine mammary tumors, most feline mammary tumors are malignant at diagnosis, and FMC is commonly characterized by high-grade histology and aggressive clinical behavior, with frequent relapse after surgery and early metastasis to regional lymph nodes, lungs, and pleura [4,142,144,145]. An important anatomical distinction is that humans have one pair of breasts, whereas cats typically have four pairs of mammary glands arranged in two longitudinal chains with drainage to the axillary and superficial inguinal lymph nodes [146,147,148,149]. Although multifocal disease also occurs in human breast cancer (HBC), cats commonly present with synchronous tumors involving multiple mammary glands; one series reported multiple masses in approximately 60% of affected cats [150,151]. Tumors within the same cat may differ in histologic type or surrogate molecular subtype, providing opportunities for within-cat comparisons but also requiring each lesion to be evaluated separately [152,153]. The comparative relevance of FMC is strongest for aggressive HBC subtypes. In human breast carcinoma, high-grade tumors are typically characterized by reduced tubule formation, marked nuclear pleomorphism, and high mitotic activity, and they may also show lymphovascular invasion and desmoplastic stroma [153,154,155,156]. FMC shares many of these aggressive histologic features, including poor differentiation, nuclear atypia, high mitotic activity, lymphovascular invasion, and stromal desmoplasia [14,28,29,142,157,158,159,160]. FMCs are also frequently hormone receptor-negative, and a substantial subset shows a triple-negative immunophenotype resembling human triple-negative breast cancer (TNBC) [28,142,153,159]. Molecular studies further support the relevance of FMC as a comparative model for aggressive HBC subtypes. Conserved oncogenic pathways are dysregulated in both FMC and human TNBC, including alterations involving *TP53*, PTEN/AKT/mTOR signaling, CXCR4-mediated metastatic pathways, and EMT programs [16,22,161,162]. These subtype-specific molecular features are discussed further in Section 3.2. Together, these clinical, histologic, and molecular similarities suggest that FMC may be useful for studying aggressive HBC subtypes, especially TNBC-like disease, rather than serving as a broad model for all forms of HBC. This comparative framework supports the use of FMC to investigate tumor progression, metastatic biology, therapeutic resistance, and emerging targeted therapeutic strategies.

### 3.1. Risk Factors

Several risk factors associated with HBC, including aging, lifetime hormonal exposure, and environmental toxicants, have also been implicated in FMC development [142,163,164,165,166,167]. In cats, the incidence of FMC increases markedly with age, with most tumors diagnosed in middle-aged to older animals, particularly between 10 and 14 years of age [168,169]. Lifetime hormonal exposure is a major risk factor for FMC, and early ovariohysterectomy markedly reduces this risk. Cats spayed before 6 months of age have an approximately 91% lower risk of developing FMC, while those spayed before 1 year of age have an approximately 86% lower risk [142,164,170]. This protective effect is thought to result primarily from reduced lifetime exposure to endogenous ovarian hormones, particularly estrogen and progesterone [142,164]. Consistently, exposure to exogenous progestogens has been associated with an increased risk of feline mammary hyperplasia and neoplasia, including in male cats [171,172]. Environmental factors may also contribute to FMC development. Heavy metals and environmental pollutants have been proposed as potential risk factors, possibly through endocrine disruption, oxidative stress, chronic inflammation, and genomic instability [173].

Breed-associated susceptibility to FMC is less consistently defined than age and hormonal risk factors [174,175]. Earlier North American studies reported an approximately two-fold increased risk of mammary carcinoma in Siamese cats [163], whereas other studies have reported different breed distributions among affected cats [174,176,177]. More recent UK primary-care data suggest an increased risk in purebred cats overall, whereas Brazilian cohorts have not identified a clear breed predisposition [174,178]. These differences likely reflect regional variation in breed distribution and the proportion of mixed-breed cats within each study population. Therefore, breed should be considered a possible population-dependent risk factor rather than a consistently established predictor of FMC risk.

Several FMC risk factors parallel epidemiological patterns observed in HBC. In women, advancing age is one of the strongest risk factors, with the highest incidence occurring after 50 years of age, and risk continues to increase with increased age [165,166,167,179,180]. Prolonged lifetime exposure to estrogen and progesterone also contributes substantially to breast cancer risk. Reproductive and hormonal factors that increase cumulative hormonal exposure are associated with increased breast cancer susceptibility, including nulliparity, late age at first pregnancy, early menarche, late menopause, and shorter duration of breastfeeding [167,179,180]. Environmental exposures may also contribute to mammary carcinogenesis. Volatile organic compounds, particulate matter, and heavy metals have been linked to endocrine disruption, chronic inflammation, oxidative stress, epigenetic changes, and altered gene-expression pathways involved in breast cancer development and progression [181,182,183,184]. Collectively, the shared influences of aging, hormonal exposure, and environmental toxicants across both species support the value of FMC as a spontaneous comparative model for investigating hormone-associated and environmentally influenced mammary carcinogenesis.

### 3.2. Molecular and Biomarker Parallels

In HBC, tumors are commonly classified using the expression of estrogen receptor (ER), progesterone receptor (PR), and human epidermal growth factor receptor 2 (HER2) [185,186]. Combined assessment of these biomarkers allows classification into clinically relevant subtypes, including hormone receptor-positive/HER2-negative (ER+/HER2−), HER2-positive, triple-positive (ER+/PR+/HER2+), and TNBC (ER−/PR−/HER2−) [185,186,187,188]. Within hormone receptor-positive disease, luminal A-like and luminal B-like tumors are further distinguished by proliferative activity, commonly assessed by Ki-67 expression, together with PR expression and HER2 status [185,186,187,188,189]. Luminal A tumors are typically ER+/PR+/HER2− with low Ki-67 expression and are generally associated with the most favorable clinical outcomes [185,186]. In contrast, luminal B-like tumors usually show higher proliferative activity, lower PR expression, and are associated with more aggressive clinical behavior [189]. TNBC lacks ER, PR, and HER2 expression and is characterized by poorly differentiated, highly proliferative, high-grade tumors. Triple-negative breast cancer comprises distinct molecular subtypes, including a basal-like subtype defined by high gene expression of basal cytokeratins such as *CK5/6*, *CK14* and *CK17* [186,190,191,192]. It remains one of the most aggressive breast cancer subtypes because of its marked biologic heterogeneity and limited availability of targeted hormonal or HER2-directed therapies [193,194,195,196,197].

Similar to HBC classification systems, feline mammary adenocarcinomas have been stratified using ER, PR, and HER2 expression into luminal-like, HER2-positive, and triple-negative subtypes. Immunohistochemistry-based studies identified six surrogate molecular subtypes analogous to HBC categories: luminal A, luminal B/HER2-negative, luminal B/HER2-positive, HER2-positive, triple-negative basal-like, and triple-negative non-basal-like [28,153,159]. Notably, FMCs are predominantly hormone receptor-negative and share important clinicopathologic, immunophenotypic, and molecular similarities with aggressive human TNBC [14,28,29,142,157,158,159,160]. One study reported that 58% of feline mammary adenocarcinomas were triple-negative [28], while another found that 85% of FMCs displayed a triple-negative phenotype [159]. Consistent with the aggressive behavior of human TNBC, triple-negative FMCs have been associated with larger tumor size, poor differentiation, necrosis, high mitotic activity, increased invasive behavior, and shorter overall survival [28,142,153,159]. Although luminal A-like and luminal B-like subtypes have been described in FMC, FMC is not yet well established as a model of hormone-responsive luminal breast cancer [175,198]. HER2-positive FMCs have also been reported, but the estimated prevalence varies by assay, antibody, and scoring criteria, with some studies reporting approximately 30% of cases with HER2 overexpression [199,200]. Overall, current evidence most strongly supports FMC as a spontaneous model of aggressive TNBC-like breast cancer rather than as a broad model for all HBC subtypes.

Integrative molecular analyses also indicate that FMC most closely resembles aggressive human TNBC rather than the full spectrum of human breast carcinoma subtypes (Table 2) [13,29]. RNA-seq profiling of FMC tumors and derived cell lines has shown dysregulation of cell-cycle, metabolic, heat-shock, p53-associated, and centrosome-regulatory programs [201]. These alterations overlap with key molecular features of aggressive TNBC, including high proliferative activity, TP53 pathway disruption, metabolic reprogramming, HSP90-associated proteostasis, and FOXM1/E2F-driven mitotic programs, all of which contribute to genomic instability, therapeutic resistance, and poor clinical outcome [202,203,204,205,206]. Copy-number and genomic sequencing studies further support this comparison. In FMC, copy-number losses in tumor-suppressor loci and gains in oncogenic loci such as *MYC* have been reported [156]. This pattern resembles human TNBC, which is characterized by extensive genomic instability, frequent copy-number alterations, gains in oncogenic regions such as *MYC*, and copy-number losses in tumor-suppressor loci [207,208,209,210]. Large-scale feline cancer sequencing studies have also identified recurrent driver-gene alterations in FMC, including *PIK3CA*, *TP53*, and *PTEN* alterations [16,22,161,162]. *PIK3CA* mutations have been reported in 22 of 47 FMCs, whereas *PTEN* alterations primarily occur through copy-number loss [16,157,211]. These alterations are biologically relevant because *PIK3CA* activation and *PTEN* loss can activate PI3K/AKT/mTOR signaling, promoting tumor-cell proliferation, survival, angiogenesis, metastatic progression, and therapeutic resistance in human TNBC [161,162,212,213]. *TP53* alterations further contribute to impaired DNA-damage responses, genomic instability, and aggressive clinical behavior, particularly in TNBC [212,214]. Together, these transcriptomic, copy-number, and genomic data support FMC as a spontaneous comparative model for studying molecular mechanisms in aggressive TNBC-like breast cancer.

Immune-checkpoint and prognostic biomarkers show several similarities between FMC and HBC. PD-1, PD-L1, and PD-L2 expression have been reported in FMC [10,215,216], similar to aggressive HBC subtypes such as TNBC, where immune-checkpoint signaling contributes to immune evasion and therapeutic response [217,218]. Bcl-2 expression also appears to have similar prognostic associations in both species. In FMC, Bcl-2 expression has been linked to longer disease-free interval and overall survival [219]. Similarly, in HBC, high Bcl-2 expression is associated with lower tumor grade, hormone receptor positivity, improved survival, and reduced tumor aggressiveness [220,221].

In addition to established molecular and immunologic markers (Table 2), tissue proteomic studies in FMC have identified discovery-stage biomarker candidates, including dermatopontin (*DPT*) and sorting nexin 5 (*SNX5*) [14,222]. Reduced *DPT* expression is associated with poorer overall survival and linked to predicted drug-response patterns involving doxorubicin, lapatinib, and neratinib, although the translational relevance of these findings requires further validation [14,222]. Collectively, these molecular and biomarker parallels support the value of FMC as a spontaneous comparative model for aggressive HBC, particularly TNBC, and provide opportunities for cross-species biomarker discovery, prognostic modeling, and development of targeted therapeutic strategies.

**Table 2:** Shared molecular features between FMC and HBC.

Molecular Feature	HBC	FMC
*TP53*	*TP53* is frequently altered in aggressive breast cancer, with mutations reported in approximately 80% of TNBC cases [223]. Loss or alteration of p53 function is associated with high-grade, poor-prognosis tumors and increased metastatic potential [224].	*TP53* mutations have been recurrently reported in FMC [16,160,225,226].
*PIK3CA*	*PIK3CA* is one of the most frequently mutated genes in breast cancer [227]. The PI3K/AKT/mTOR pathway promotes tumor-cell growth, survival, and therapeutic vulnerability [227,228,229,230]. Activating mutations are reported in ~26% of invasive breast carcinomas overall and 13–22.1% of TNBC [231,232].	*PIK3CA* has been identified as an FMC driver, with mutations reported in approximately 45% of FMCs [16,233].
*PTEN*	*PTEN* loss promotes proliferation, invasion, metastatic competence, and treatment resistance, and has been detected in approximately 48.6% of TNBC cases in some cohorts [234,235].	*PTEN* is a recurrent FMC driver. Loss of PTEN expression is reported in ~77% of cases and has been associated with poorer prognosis and shorter survival [16,157,211].
*FBXW7*	FBXW7 is a tumor suppressor, and low FBXW7 expression has been associated with poorer prognosis in breast cancer patients [236,237,238].	*FBXW7* has been identified as an FMC driver, with mutations reported in approximately 72% of cases [16].
CXCL12/CXCR4	Overexpression of the CXCL12/CXCR4 axis promotes tumor growth, metastasis, and chemotherapy resistance, particularly in aggressive breast cancer, including TNBC [239,240,241].	Serum SDF-1/CXCL12 has been proposed as a diagnostic biomarker in FMC; CXCR4 overexpression was detected in 86% of tumors [242].
Leptin/ObR	In HBC, leptin–ObR signaling promotes tumor-cell survival, angiogenesis, inflammation, and disease progression, particularly in obesity-associated breast cancer biology [243,244]. Leptin and ObR expression were detected in 83% and 33.7% of breast cancer tissues, respectively [245].	ObR is overexpressed in FMC tissues, and serum ObR levels have been correlated with immune downregulation and tumor development [10,29,246].
Immune checkpoint	In HBC, especially TNBC, the PD-1/PD-L1 immune-checkpoint pathway can suppress antitumor immune responses [217,218]. Tumoral PD-L1 staining was reported in 12% of breast cancers overall and 32% of triple-negative cases [247].	PD-1 and PD-L1 are detectable in FMC, with enrichment reported in aggressive subtypes, including HER2-positive and triple-negative tumors [10,215,216,248].
*Bcl-2*	In HBC, high Bcl-2 expression is associated with a favorable prognosis, improved survival, and less aggressive disease [220,221].	Bcl-2 expression has been associated with longer disease-free interval and overall survival in FMC [219].

FMC, feline mammary carcinoma; TNBC, triple-negative breast cancer; HER2, human epidermal growth factor receptor 2; ObR, leptin receptor; PD-1, programmed cell death protein 1; PD-L1, programmed death-ligand 1.

### 3.3. Tumor Microenvironment Parallels

The tumor microenvironment plays an important role in breast cancer progression, therapeutic resistance, and immune evasion in both humans and cats. In HBC, particularly in TNBC, extracellular matrix remodeling, cancer-associated fibroblasts (CAFs), regulatory T-cell enrichment, leptin signaling, and reduced cytotoxic T-cell infiltration have been associated with invasion, metastatic progression, immune suppression, and poor clinical outcome [249,250]. Similar features have been reported in FMC, including collagen remodeling, CAF enrichment, increased regulatory T-cell infiltration, and enhanced leptin signaling, all of which correlate with more aggressive tumor behavior and reduced survival [10,246,251,252,253,254].

Recent spatial immunohistochemical analyses further support similarities in immune and stromal organization between FMC and aggressive HBC. One immunohistochemical study found that high-grade FMC is characterized by an immunosuppressed, highly vascularized, and highly proliferative tumor microenvironment [9]. In high-grade FMC, macrophages, neutrophils, and antigen-presenting cells were predominantly localized within the tumor-associated stroma surrounding neoplastic cell nests, whereas intratumoral T cells demonstrated limited activation [9,10]. This stromal immune architecture may support tumor progression by promoting angiogenesis, extracellular matrix remodeling, invasion, and suppressing effective antitumor immune responses [9]. Similar features are observed in aggressive HBC, particularly TNBC, where stromal immune exclusion, tumor-associated macrophage enrichment, and limited CD8+ T-cell infiltration into tumor-cell clusters are associated with invasion, immune evasion, and adverse clinical behavior [255,256].

Several pathways involved in tumor–stromal communication also appear to be shared between FMC and HBC. For example, CXCL12–CXCR4 signaling axis promotes tumor-cell migration, metastatic spread, angiogenesis, and formation of a supportive tumor microenvironment in HBC [257]. This pathway has also been implicated in FMC, where serum CXCL12 has been proposed as a potential diagnostic biomarker in HER2-overexpressing feline mammary tumors [241,242]. Leptin signaling represents another shared pathway. In HBC, leptin–leptin receptor (ObR) signaling promotes tumor-cell survival, angiogenesis, proliferation, inflammation, and disease progression, particularly in obesity-associated breast cancer biology [243,244]. Overexpression of ObR has also been reported in FMC [10,29].

A recent review summarized emerging evidence that immune-checkpoint expression, inflammatory and adipokine signaling, chemokine alterations, and angiogenic pathways contribute to tumor–microenvironment interactions in FMC [258]. These mechanisms are also relevant to aggressive HBC, including TNBC, where immune infiltration, PD-1/PD-L1 signaling, macrophage polarization, stromal remodeling, and angiogenesis influence prognosis and treatment response [259,260,261]. Together, these findings suggest that FMC shares several stromal and immune features with aggressive HBC, particularly TNBC. This supports the use of FMC as a comparative model for studying tumor–stromal interactions, immune escape, and microenvironment-targeted therapeutic strategies in an immunocompetent host.

### 3.4. Patient-Derived FMC Organoids

The comparative value of spontaneous FMC can be further strengthened by emerging patient-derived experimental platforms. A large-scale feline oncogenomics study used patient-derived FMC tumoroids to test whether genomic alterations could predict drug sensitivity [16]. *FBXW7* was identified as a frequent candidate driver in FMC, with mutations reported in approximately 53–72% of cases across sequencing cohorts [16]. Because FBXW7 functions as a tumor suppressor that promotes degradation of cell-cycle and mitotic regulators, its mutation may create vulnerability to drugs that disrupt mitosis [237,238]. Consistent with this hypothesis, *FBXW7*-mutant feline mammary tumoroids were significantly more sensitive than *FBXW7*-wild-type tumoroids to microtubule-disrupting chemotherapies [16]. Notably, recurrent *FBXW7* driver alterations in FMC overlap with genomic alterations reported for patient selection in human precision oncology trials [262], suggesting that feline genomic profiling can identify human-relevant therapeutic hypotheses for functional testing in patient-derived tumoroids. A separate organoid study further supported the translational value of FMC organoids by establishing patient-derived models that recapitulated tumor morphology, receptor heterogeneity, tumorigenic potential, and variable drug sensitivity [14]. Using this platform, the authors identified the LMTK3/FADS2 axis as a candidate pathway involved in FMC proliferation, invasion, and apoptosis resistance [14]. LMTK3 inhibition reduced FMC organoid viability, inhibited invasion, induced apoptosis, and suppressed tumor growth in xenograft models [14]. Importantly, LMTK3 inhibition also reduced the viability of HBC organoids, supporting the LMTK3/FADS2 axis as a potentially conserved progression pathway across feline and HBC [14]. Together, these studies show that patient-derived FMC organoids can connect genomic alterations with functional drug response, identify candidate therapeutic vulnerabilities, and prioritize conserved targets for further validation in HBC models.

### 3.5. FMC Therapeutic Studies

Therapeutic studies in FMC have evaluated conventional cytotoxic chemotherapy, targeted therapies, and emerging molecular approaches, providing translational insights relevant to HBC, particularly TNBC. Most clinical studies of cytotoxic chemotherapy in cats have focused on adjuvant doxorubicin after surgical resection. In one study, cats treated with surgery followed by doxorubicin had a disease-free interval of 255 days and a median survival time of 448 days [263]. However, a subsequent multi-institutional comparison of adjuvant doxorubicin, metronomic cyclophosphamide plus meloxicam, and surgery alone found no significant survival benefit for either chemotherapy regimen over surgery alone in unselected cats with FMC [264,265]. These findings highlight the need for biomarker-driven treatment stratification.

Targeted therapy studies in FMC have primarily focused on HER2- and PI3K/AKT/mTOR-related pathways. In vitro studies of anti-HER2 monoclonal antibodies and HER2-targeted tyrosine kinase inhibitors, including lapatinib and neratinib, have demonstrated dose-dependent antiproliferative effects across FMC cell lines [266]. Synergistic activity has also been observed when these tyrosine kinase inhibitors were combined with the mTOR inhibitor rapamycin [266]. Together, these findings support further investigation of HER2- and PI3K/AKT/mTOR-directed strategies in cats with spontaneous FMC.

To date, no published studies have evaluated PD-1/PD-L1 checkpoint blockade as a therapeutic intervention in cats with spontaneous FMC. However, the PD-1/PD-L1 pathway has emerged as a promising immunotherapeutic target based on studies demonstrating its expression and biologic relevance in FMC [216,258]. A more directly translational example comes from a case report evaluating an iron oxide nanoparticle-delivered miR-10b inhibitor in a cat with spontaneous metastatic FMC [267]. Magnetic resonance imaging confirmed tumor delivery, and molecular analyses demonstrated on-target engagement, providing proof-of-concept evidence to support progression toward human clinical trials [267]. Nevertheless, no prospective phase I or II clinical trials in cats with spontaneous FMC have yet been completed with the mechanistic rigor typical of comparative oncology trial design. As a result, the current therapeutic evidence base for FMC remains largely retrospective, preclinical, or limited to small translational studies.

## 4. Current Challenges and Future Directions

Spontaneous feline cancers are increasingly recognized as valuable comparative oncology models because they share biologic and clinical features with human cancers that are difficult to reproduce faithfully in ex vivo systems and conventional rodent models [2,3,16]. Previous reviews have established the broader value of cats in comparative oncology and have summarized key clinical, pathologic, and molecular features of FOSCC and FMC [32,35,142]. Building on this foundation, this review compares FOSCC and FMC with their corresponding human cancers across epidemiology, histology, molecular pathways, tumor microenvironment features, therapeutic studies, and emerging model systems. Recent genomic, transcriptomic, and tumor microenvironment studies support the translational relevance of FOSCC for HNSCC and FMC for aggressive TNBC [4,16,26,34]. These shared features provide opportunities for biomarker discovery, therapeutic testing, and improved cancer care in both cats and humans.

Although spontaneous feline tumors offer important strengths for comparative oncology, they also have limitations. Compared with laboratory models, studies of spontaneous feline tumors have less control over genetic background, environmental exposures, reproductive history, prior treatment, biospecimen handling, and longitudinal follow-up. Therefore, spontaneous feline tumors should be viewed as complementary to, rather than replacements for, laboratory models. A major limitation of the current feline comparative oncology literature is heterogeneity across study populations and methods. Differences in cohort composition, tumor type, breed distribution, reproductive status, clinical management, sample processing, and follow-up can make direct comparisons across studies difficult and may weaken cross-species conclusions. Variation in classification and grading systems is another important challenge. Some studies use traditional veterinary histopathologic criteria, whereas others adapt human cancer classification frameworks. For example, p16, pRb, and p53 staining patterns in FOSCC may differ from those typically described in human HNSCC, highlighting the need to validate human-derived biomarker frameworks before applying them to feline disease [119]. Similarly, FMC studies use inconsistent grading systems. Some rely on traditional veterinary histologic grading systems, such as the World Health Organization-based approach used by Millanta et al. [268], whereas others apply HBC-inspired molecular subtyping based on markers such as ER, PR, HER2, Ki-67, and EGFR [215]. This lack of standardization may affect subtype assignment, prognostic interpretation, and comparisons between feline and human mammary tumors. These limitations do not diminish the value of spontaneous feline tumors, but they highlight the need for more standardized study designs.

Future studies should prioritize larger multicenter cohorts, prospective enrollment, and appropriately matched tumor and control groups. Key clinical, demographic, and biospecimen variables should be recorded systematically, including age, breed, sex, reproductive status, age at neutering, geographic region, tumor site, stage, prior treatment, biospecimen type, and clinical outcome. Careful documentation of these variables is essential because uncontrolled variation can obscure true biologic associations and limit comparisons across studies. Standardized biospecimen collection, centralized pathology review, harmonized grading and classification criteria, validated feline-specific or cross-reactive antibodies, and consistent molecular workflows are particularly important for genomic, transcriptomic, immune, and tumor microenvironment studies. Prospective feline clinical trials should further incorporate molecular stratification, standardized response criteria, toxicity assessment, quality-of-life measures, disease-free interval, and overall survival. Together, these approaches would improve reproducibility, strengthen cross-species interpretation, and help define which feline tumors are most suitable as translational models for specific human cancer subtypes.

## Data Availability

No new data were created or analyzed in this study. Data sharing is not applicable to this article.

## Abbreviations

The following abbreviations are used in this manuscript:

AKT	Protein kinase B
Bcl-2	B-cell lymphoma 2
CAF	Cancer-associated fibroblast
CDKN1A	Cyclin-dependent kinase inhibitor 1A
COX-2	Cyclooxygenase-2
CS3D	Cyclic STAT3 decoy oligonucleotide
CXCL12	C-X-C motif chemokine ligand 12
CXCR4	C-X-C chemokine receptor type 4
DFMO	Difluoromethylornithine
DNA	Deoxyribonucleic acid
DPT	Dermatopontin
EGFR	Epidermal growth factor receptor
EMT	Epithelial-to-mesenchymal transition
ER	Estrogen receptor
FADS2	Fatty acid desaturase 2
FAS	Fas cell surface death receptor
FBXW7	F-box and WD repeat domain containing 7
FcaPV	*Felis catus* papillomavirus
FMC	Feline mammary carcinoma
FOSCC	Feline oral squamous cell carcinoma
FOXM1	Forkhead box M1
HBC	Human breast cancer
HER2	Human epidermal growth factor receptor 2
HNSCC	Head and neck squamous cell carcinoma
HPV	Human papillomavirus
IL-6	Interleukin-6
JAK	Janus kinase
LMTK3	Lemur tyrosine kinase 3
mTOR	Mechanistic target of rapamycin
MYC	MYC proto-oncogene, bHLH transcription factor
NF-κB	Nuclear factor kappa B
ObR	Leptin receptor
PCR	Polymerase chain reaction
PD-1	Programmed cell death protein 1
PD-L1	Programmed death-ligand 1
PD-L2	Programmed death-ligand 2
PI3K	Phosphoinositide 3-kinase
PIK3CA	Phosphatidylinositol-4,5-bisphosphate 3-kinase catalytic subunit alpha
PR	Progesterone receptor
PTEN	Phosphatase and tensin homolog
SCC	Squamous cell carcinoma
SDF-1	Stromal cell-derived factor 1
SNX5	Sorting nexin 5
STAT3	Signal transducer and activator of transcription 3
TNBC	Triple-negative breast cancer
TNF	Tumor necrosis factor
TP53	Tumor protein p53
TWIST1	Twist family bHLH transcription factor 1
VEGF	Vascular endothelial growth factor
WNT	Wingless/Int-1 signaling pathway
ZEB1	Zinc finger E-box binding homeobox 1
